# Supplementary material for: Classical Swine Fever Virus Biology, Clinicopathology, Diagnosis, Vaccines and a Meta-Analysis of Prevalence: A Review from the Indian Perspective
Source: Pathogens. 2020 Jun 22;9(6):500. doi: 10.3390/pathogens9060500 (PMC7350356; doi:10.3390/pathogens9060500)
Supplement: Supplementary file 1 [file pathogens-09-00500-s001.pdf]

Study	Events	Total	Proportion	95%−CI	Weight (fixed)	Weight (random)
PDADMAS 2011	18	100	0.18	[0.11; 0.26]	0.7%	1.4%
PDADMAS 2011	15	125	0.12	[0.07; 0.18]	0.9%	1.4%
PDADMAS 2011	43	100	0.43	[0.33; 0.53]	0.7%	1.4%
PDADMAS 2011	81	844	0.10	[0.08; 0.12]	5.8%	1.4%
PDADMAS 2011	34	100	0.34	[0.25; 0.44]	0.7%	1.4%
PDADMAS 2014	5	65	0.08	[0.02; 0.16]	0.5%	1.3%
PDADMAS 2012	51	58	0.88	[0.78; 0.95]	0.4%	1.3%
Ahuja et al 2014	138	264	0.52	[0.46; 0.58]	1.8%	1.4%
Ahuja et al 2014	97	252	0.38	[0.33; 0.45]	1.7%	1.4%
Bhaskar et al 2015	6	19	0.32	[0.12; 0.55]	0.1%	1.2%
Choori et al 2015	89	218	0.41	[0.34; 0.47]	1.5%	1.4%
Lalthaswammawii 2008	20	350	0.06	[0.03; 0.08]	2.4%	1.4%
Mahajan et al 2011	18	49	0.37	[0.24; 0.51]	0.3%	1.3%
Mukherjee et al 2018*	382	500	0.76	[0.73; 0.80]	3.4%	1.4%
Mukherjee et al 2018*	193	292	0.66	[0.61; 0.71]	2.0%	1.4%
Mukherjee et al 2018*	282	1106	0.25	[0.23; 0.28]	7.6%	1.4%
Nandi et al 2011	10	11	0.91	[0.65; 1.00]	0.1%	1.1%
Nandi et al 2011	126	171	0.74	[0.67; 0.80]	1.2%	1.4%
Nandi et al 2011	40	46	0.87	[0.75; 0.95]	0.3%	1.3%
Nandi et al 2011	157	296	0.53	[0.47; 0.59]	2.0%	1.4%
Nandi et al 2011	43	70	0.61	[0.50; 0.73]	0.5%	1.4%
Nandi et al 2011	118	147	0.80	[0.73; 0.86]	1.0%	1.4%
NIVEDI 2015	54	1120	0.05	[0.04; 0.06]	7.7%	1.4%
NIVEDI 2015	38	100	0.38	[0.29; 0.48]	0.7%	1.4%
PDADMAS 2014	15	36	0.42	[0.26; 0.58]	0.3%	1.3%
PDADMAS 2014	25	87	0.29	[0.20; 0.39]	0.6%	1.4%
PDADMAS 2014	16	51	0.31	[0.19; 0.45]	0.4%	1.3%
PDADMAS 2014	16	43	0.37	[0.23; 0.52]	0.3%	1.3%
PDADMAS 2014	35	52	0.67	[0.54; 0.79]	0.4%	1.3%
PDADMAS 2014	22	46	0.48	[0.33; 0.62]	0.3%	1.3%
PDADMAS 2014	31	58	0.53	[0.40; 0.66]	0.4%	1.3%
NIVEDI 2015	1	5	0.20	[0.00; 0.68]	0.0%	0.9%
NIVEDI 2015	1	1	1.00	[0.00; 1.00]	0.0%	0.5%
NIVEDI 2015	0	2	0.00	[0.00; 0.70]	0.0%	0.7%
NIVEDI 2015	0	1	0.00	[0.00; 1.00]	0.0%	0.5%
NIVEDI 2015	15	25	0.60	[0.40; 0.79]	0.2%	1.3%
NIVEDI 2015	11	61	0.18	[0.09; 0.29]	0.4%	1.3%
NIVEDI 2015	0	27	0.00	[0.00; 0.06]	0.2%	1.3%
NIVEDI 2015	2	8	0.25	[0.01; 0.62]	0.1%	1.1%
NIVEDI 2015	2	29	0.07	[0.00; 0.20]	0.2%	1.3%
NIVEDI 2015	26	100	0.26	[0.18; 0.35]	0.7%	1.4%
NIVEDI 2016	66	105	0.63	[0.53; 0.72]	0.7%	1.4%
NIVEDI 2016	44	59	0.75	[0.63; 0.85]	0.4%	1.3%
NIVEDI 2016	142	192	0.74	[0.67; 0.80]	1.3%	1.4%
NIVEDI 2016	7	9	0.78	[0.44; 0.99]	0.1%	1.1%
NIVEDI 2016	0	2	0.00	[0.00; 0.70]	0.0%	0.7%
NIVEDI 2016	0	2	0.00	[0.00; 0.70]	0.0%	0.7%
NIVEDI 2017	15	73	0.21	[0.12; 0.31]	0.5%	1.4%
NIVEDI 2017	189	509	0.37	[0.33; 0.41]	3.5%	1.4%
NIVEDI 2017	65	304	0.21	[0.17; 0.26]	2.1%	1.4%
NIVEDI 2017	119	270	0.44	[0.38; 0.50]	1.9%	1.4%
NIVEDI 2017	26	72	0.36	[0.25; 0.48]	0.5%	1.4%
NIVEDI 2017	11	104	0.11	[0.05; 0.17]	0.7%	1.4%
NIVEDI 2017	510	652	0.78	[0.75; 0.81]	4.5%	1.4%
NIVEDI 2018	0	25	0.00	[0.00; 0.07]	0.2%	1.3%
NIVEDI 2018	17	35	0.49	[0.32; 0.65]	0.2%	1.3%
NIVEDI 2018	2	8	0.25	[0.01; 0.62]	0.1%	1.1%
NIVEDI 2018	0	10	0.00	[0.00; 0.17]	0.1%	1.1%
NIVEDI 2018	0	36	0.00	[0.00; 0.05]	0.3%	1.3%
NIVEDI 2018	0	36	0.00	[0.00; 0.05]	0.3%	1.3%
NIVEDI 2019	668	1060	0.63	[0.60; 0.66]	7.3%	1.4%
PDADMAS 2011	126	498	0.25	[0.22; 0.29]	3.4%	1.4%
PDADMAS 2011	63	510	0.12	[0.10; 0.15]	3.5%	1.4%
PDADMAS 2011	36	166	0.22	[0.16; 0.28]	1.1%	1.4%
PDADMAS 2011	12	83	0.14	[0.08; 0.23]	0.6%	1.4%
PDADMAS 2012	0	9	0.00	[0.00; 0.18]	0.1%	1.1%
PDADMAS 2012	16	21	0.76	[0.55; 0.92]	0.1%	1.2%
PDADMAS 2012	0	5	0.00	[0.00; 0.32]	0.0%	0.9%
PDADMAS 2012	22	64	0.34	[0.23; 0.47]	0.4%	1.3%
PDADMAS 2012	10	22	0.45	[0.25; 0.67]	0.2%	1.2%
PDADMAS 2012	111	254	0.44	[0.38; 0.50]	1.8%	1.4%
PDADMAS 2012	32	51	0.63	[0.49; 0.76]	0.4%	1.3%
Rajbongshi 2015	186	325	0.57	[0.52; 0.63]	2.2%	1.4%
Raut et al 2015	54	1120	0.05	[0.04; 0.06]	7.7%	1.4%
Rout et al 2013	65	110	0.59	[0.50; 0.68]	0.8%	1.4%
Sarma et al 2008	4	7	0.57	[0.19; 0.92]	0.1%	1.0%
Sarma et al 2008	7	7	1.00	[0.77; 1.00]	0.1%	1.0%
Shivaraj et al 2013	173	517	0.33	[0.29; 0.38]	3.6%	1.4%
Shivaraj et al 2013	20	113	0.18	[0.11; 0.25]	0.8%	1.4%
**Fixed effect model**		**14480**	**0.31**	**[0.31; 0.32]**	**100.0%**	**--**
**Random effects model**			**0.35**	**[0.29; 0.43]**	**--**	**100.0%**

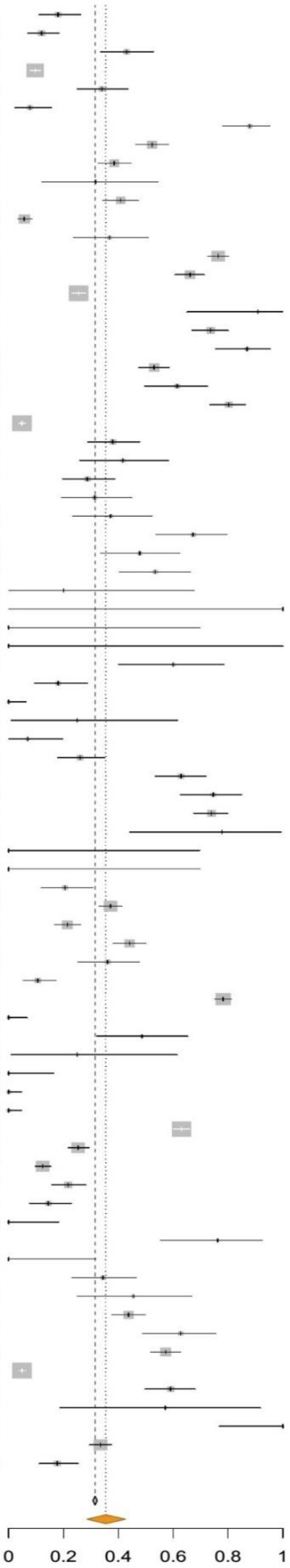

Heterogeneity: $I^2 = 98\%$, $\tau^2 = 0.0869$, $p = 0$

S1: Forest plot showing the prevalence of CSF in India

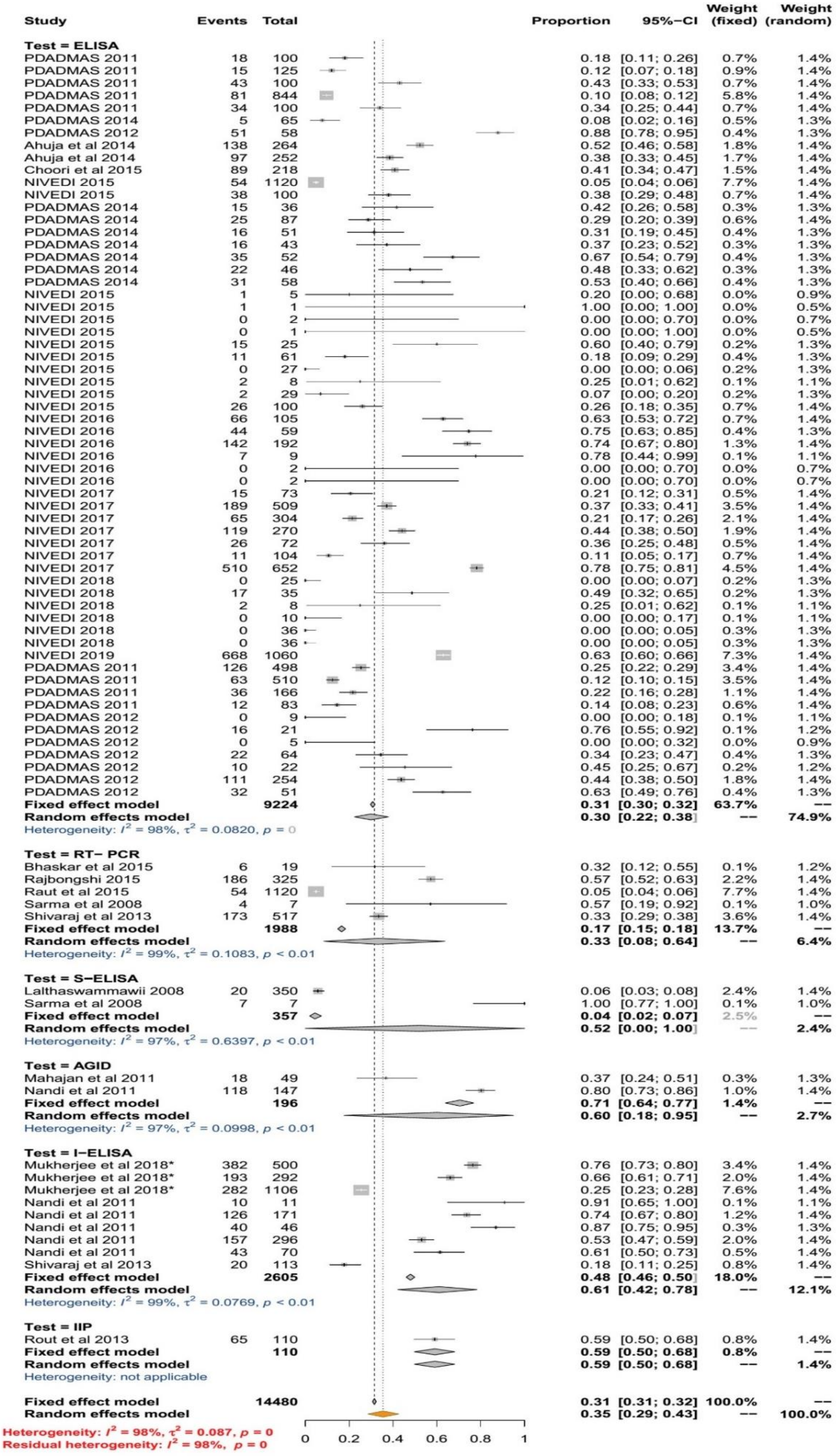

S2. Forest plot showing the diagnostic test wise prevalence of CSF in India

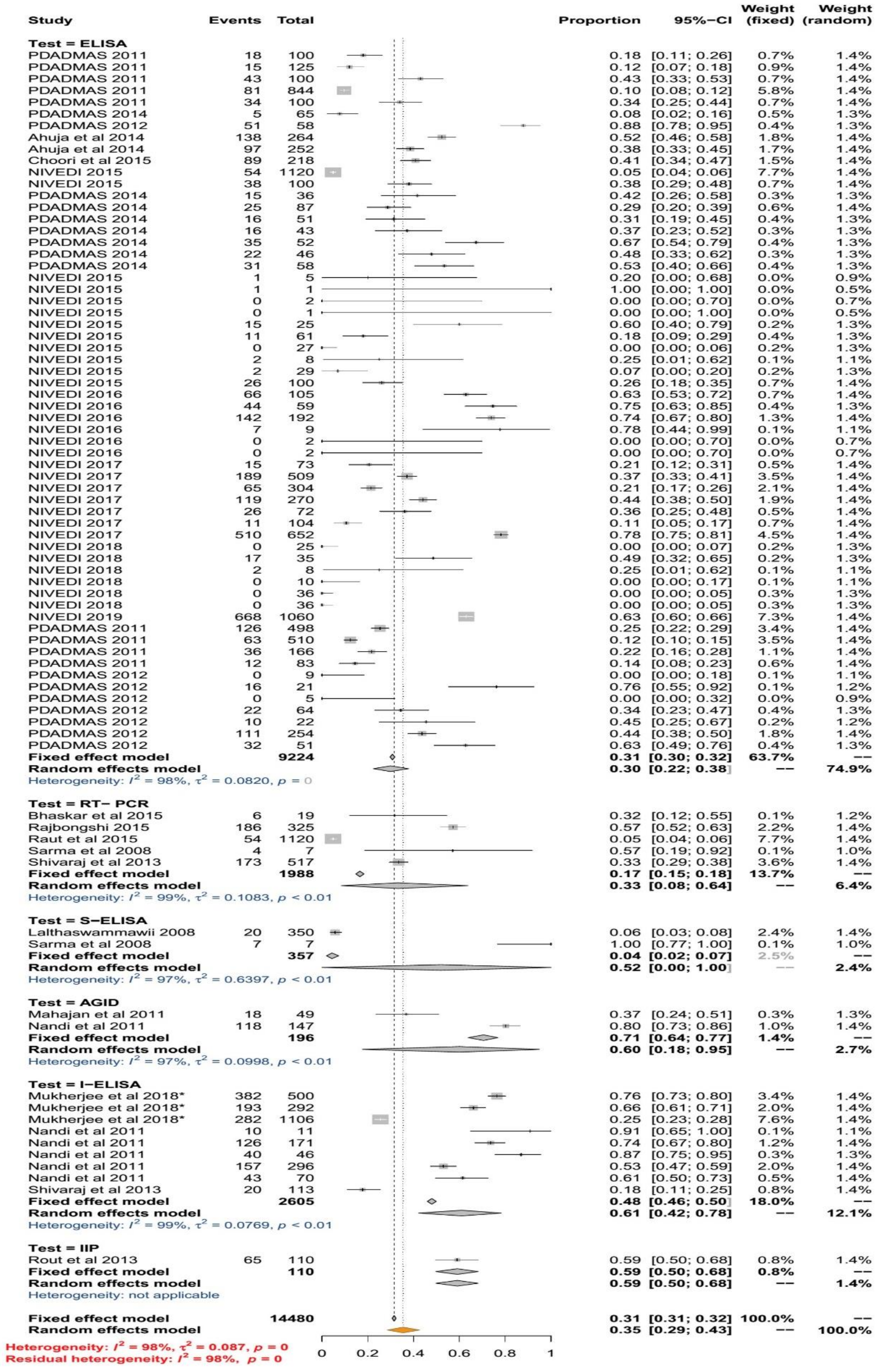

S3. Forest plot showing the region wise prevalence of CSF in India

Study	Events	Total	Proportion	95%−CI	Weight (fixed)	Weight (random)
**Yearcat = 2011−15**						
PDADMAS 2011	18	100	0.18	[0.11; 0.26]	0.7%	1.4%
PDADMAS 2011	15	125	0.12	[0.07; 0.18]	0.9%	1.4%
PDADMAS 2011	43	100	0.43	[0.33; 0.53]	0.7%	1.4%
PDADMAS 2011	81	844	0.10	[0.08; 0.12]	5.8%	1.4%
PDADMAS 2011	34	100	0.34	[0.25; 0.44]	0.7%	1.4%
PDADMAS 2014	5	65	0.08	[0.02; 0.16]	0.5%	1.3%
PDADMAS 2012	51	58	0.88	[0.78; 0.95]	0.4%	1.3%
Ahuja et al 2014	138	264	0.52	[0.46; 0.58]	1.8%	1.4%
Ahuja et al 2014	97	252	0.38	[0.33; 0.45]	1.7%	1.4%
Bhaskar et al 2015	6	19	0.32	[0.12; 0.55]	0.1%	1.2%
Choori et al 2015	89	218	0.41	[0.34; 0.47]	1.5%	1.4%
Lalthaswammawii 2008	20	350	0.06	[0.03; 0.08]	2.4%	1.4%
Mahajan et al 2011	18	49	0.37	[0.24; 0.51]	0.3%	1.3%
Nandi et al 2011	10	11	0.91	[0.65; 1.00]	0.1%	1.1%
Nandi et al 2011	126	171	0.74	[0.67; 0.80]	1.2%	1.4%
Nandi et al 2011	40	46	0.87	[0.75; 0.95]	0.3%	1.3%
Nandi et al 2011	157	296	0.53	[0.47; 0.59]	2.0%	1.4%
Nandi et al 2011	43	70	0.61	[0.50; 0.73]	0.5%	1.4%
Nandi et al 2011	118	147	0.80	[0.73; 0.86]	1.0%	1.4%
NIVEDI 2015	54	1120	0.05	[0.04; 0.06]	7.7%	1.4%
NIVEDI 2015	38	100	0.38	[0.29; 0.48]	0.7%	1.4%
PDADMAS 2014	15	36	0.42	[0.26; 0.58]	0.3%	1.3%
PDADMAS 2014	25	87	0.29	[0.20; 0.39]	0.6%	1.4%
PDADMAS 2014	16	51	0.31	[0.19; 0.45]	0.4%	1.3%
PDADMAS 2014	16	43	0.37	[0.23; 0.52]	0.3%	1.3%
PDADMAS 2014	35	52	0.67	[0.54; 0.79]	0.4%	1.3%
PDADMAS 2014	22	46	0.48	[0.33; 0.62]	0.3%	1.3%
PDADMAS 2014	31	58	0.53	[0.40; 0.66]	0.4%	1.3%
NIVEDI 2015	1	5	0.20	[0.00; 0.68]	0.0%	0.9%
NIVEDI 2015	1	1	1.00	[0.00; 1.00]	0.0%	0.5%
NIVEDI 2015	0	2	0.00	[0.00; 0.70]	0.0%	0.7%
NIVEDI 2015	0	1	0.00	[0.00; 1.00]	0.0%	0.5%
NIVEDI 2015	15	25	0.60	[0.40; 0.79]	0.2%	1.3%
NIVEDI 2015	11	61	0.18	[0.09; 0.29]	0.4%	1.3%
NIVEDI 2015	0	27	0.00	[0.00; 0.06]	0.2%	1.3%
NIVEDI 2015	2	8	0.25	[0.01; 0.62]	0.1%	1.1%
NIVEDI 2015	2	29	0.07	[0.00; 0.20]	0.2%	1.3%
NIVEDI 2015	26	100	0.26	[0.18; 0.35]	0.7%	1.4%
PDADMAS 2011	126	498	0.25	[0.22; 0.29]	3.4%	1.4%
PDADMAS 2011	63	510	0.12	[0.10; 0.15]	3.5%	1.4%
PDADMAS 2011	36	166	0.22	[0.16; 0.28]	1.1%	1.4%
PDADMAS 2011	12	83	0.14	[0.08; 0.23]	0.6%	1.4%
PDADMAS 2012	0	9	0.00	[0.00; 0.18]	0.1%	1.1%
PDADMAS 2012	16	21	0.76	[0.55; 0.92]	0.1%	1.2%
PDADMAS 2012	0	5	0.00	[0.00; 0.32]	0.0%	0.9%
PDADMAS 2012	22	64	0.34	[0.23; 0.47]	0.4%	1.3%
PDADMAS 2012	10	22	0.45	[0.25; 0.67]	0.2%	1.2%
PDADMAS 2012	111	254	0.44	[0.38; 0.50]	1.8%	1.4%
PDADMAS 2012	32	51	0.63	[0.49; 0.76]	0.4%	1.3%
Rajbongshi 2015	186	325	0.57	[0.52; 0.63]	2.2%	1.4%
Raut et al 2015	54	1120	0.05	[0.04; 0.06]	7.7%	1.4%
Rout et al 2013	65	110	0.59	[0.50; 0.68]	0.8%	1.4%
Sarma et al 2008	4	7	0.57	[0.19; 0.92]	0.1%	1.0%
Sarma et al 2008	7	7	1.00	[0.77; 1.00]	0.1%	1.0%
Shivaraj et al 2013	173	517	0.33	[0.29; 0.38]	3.6%	1.4%
Shivaraj et al 2013	20	113	0.18	[0.11; 0.25]	0.8%	1.4%
**Fixed effect model**		**9019**	**0.21**	**[0.20; 0.22]**	**62.3%**	**--**
**Random effects model**			**0.36**	**[0.28; 0.43]**	**--**	**70.9%**
Heterogeneity: I^2 = 98%, τ^2 = 0.0725, p = 0						
**Yearcat = 2016−19**						
Mukherjee et al 2018*	382	500	0.76	[0.73; 0.80]	3.4%	1.4%
Mukherjee et al 2018*	193	292	0.66	[0.61; 0.71]	2.0%	1.4%
Mukherjee et al 2018*	282	1106	0.25	[0.23; 0.28]	7.6%	1.4%
NIVEDI 2016	66	105	0.63	[0.53; 0.72]	0.7%	1.4%
NIVEDI 2016	44	59	0.75	[0.63; 0.85]	0.4%	1.3%
NIVEDI 2016	142	192	0.74	[0.67; 0.80]	1.3%	1.4%
NIVEDI 2016	7	9	0.78	[0.44; 0.99]	0.1%	1.1%
NIVEDI 2016	0	2	0.00	[0.00; 0.70]	0.0%	0.7%
NIVEDI 2016	0	2	0.00	[0.00; 0.70]	0.0%	0.7%
NIVEDI 2017	15	73	0.21	[0.12; 0.31]	0.5%	1.4%
NIVEDI 2017	189	509	0.37	[0.33; 0.41]	3.5%	1.4%
NIVEDI 2017	65	304	0.21	[0.17; 0.26]	2.1%	1.4%
NIVEDI 2017	119	270	0.44	[0.38; 0.50]	1.9%	1.4%
NIVEDI 2017	26	72	0.36	[0.25; 0.48]	0.5%	1.4%
NIVEDI 2017	11	104	0.11	[0.05; 0.17]	0.7%	1.4%
NIVEDI 2017	510	652	0.78	[0.75; 0.81]	4.5%	1.4%
NIVEDI 2018	0	25	0.00	[0.00; 0.07]	0.2%	1.3%
NIVEDI 2018	17	35	0.49	[0.32; 0.65]	0.2%	1.3%
NIVEDI 2018	2	8	0.25	[0.01; 0.62]	0.1%	1.1%
NIVEDI 2018	0	10	0.00	[0.00; 0.17]	0.1%	1.1%
NIVEDI 2018	0	36	0.00	[0.00; 0.05]	0.3%	1.3%
NIVEDI 2018	0	36	0.00	[0.00; 0.05]	0.3%	1.3%
NIVEDI 2019	668	1060	0.63	[0.60; 0.66]	7.3%	1.4%
**Fixed effect model**		**5461**	**0.50**	**[0.48; 0.51]**	**37.7%**	**--**
**Random effects model**			**0.35**	**[0.24; 0.47]**	**--**	**29.1%**
Heterogeneity: I^2 = 98%, τ^2 = 0.0684, p < 0.01						
**Fixed effect model**		**14480**	**0.31**	**[0.31; 0.32]**	**100.0%**	**--**
**Random effects model**			**0.35**	**[0.29; 0.43]**	**--**	**100.0%**

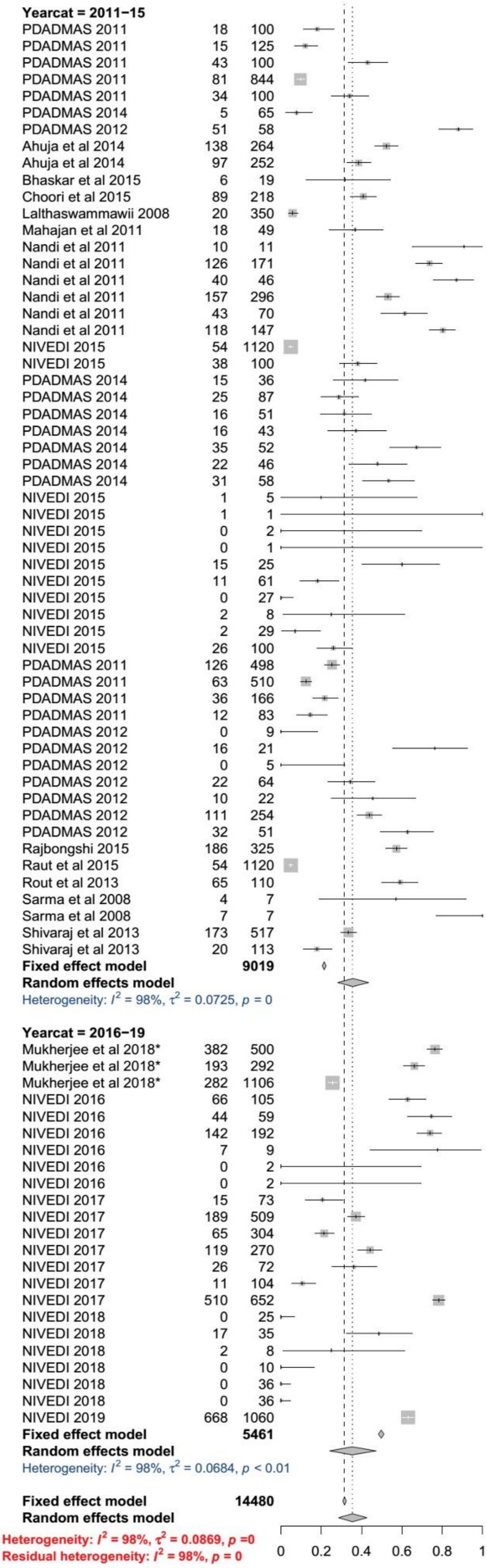

Heterogeneity: I^2 = 98%, τ^2 = 0.0869, p =0
Residual heterogeneity: I^2 = 98%, p = 0

S4. Forest plot showing the year wise prevalence of CSF in India
